# Bibliographical

**Published:** 1873

**Authors:** 


### BIBLIOGRAPHICAL.

*Contributions to Practical Surgery.* By GEORGE W. NORRIS, M.D., late Surgeon to the Pennsylvania Hospital; Vice President of the College of Physicians of Philadelphia; Member of the Société Medicale d'Observation of Paris, etc. Philadelphia: LINDSAY & BLAKISTON. 1873.

This valuable work of Dr. Norris consists of seven essays upon important surgical subjects, with numerous clinical histories, drawn from a hospital service of thirty years, and is truly a contribution to practical surgery. The statistical method of investigating the subjects considered in his essays, is certainly the most valuable way of arriving at anything like reliable conclusions. That many objections may be urged to this mode of arriving at results, we do not deny; a most prominent objection being the fact that in surgery we are, unhappily, too prone to silence in regard to our unfortunate cases, and rarely neglect to make prominent successful results after any operation at all out of the common course. At the present day, however, no one will deny the importance of statistical analysis, cautiously made, as one of the very best modes of arriving at facts upon which a sure improvement of our science is to be made.

The first chapter of the work is an exhaustive essay of one hundred and twelve pages, "*On the Occurrence of Non-union after Fractures—Its Causes and Treatment.*" By the statistics of public institutions, as well as by the private practice of surgeons, he demonstrates that this accident is much less frequent than is generally supposed,—that at the Pennsylvania Hospital, from 1830 to 1850, ten thousand one hundred and ninety-five cases of recent fracture were received, and in no one instance did non-union or false joint follow the treatment pursued. That in all the cases of false joint (eighteen in number), treated in that hospital during the twenty years above mentioned, were admitted as such.

After discussing the causes, both local and constitutional, and the various plans of treatment with reference to, or reports of, many individual cases, he gives the following *rèsumè*:

"From all that has been observed in the preceding pages upon the treatment of ununited fractures, it will be seen that we recommend:

1. To apply the method of cure by rest and compression. If the fracture has been regularly treated, and is not consolidated at the usual period, replace the limb in the apparatus, and insure to it a state of complete immovability: if the treatment of the injury has been altogether neglected, or been inefficient, apply proper splints and moderate compression with a roller, and renew these as soon as they become in any degree lax.

2. If, from want of action in the seat of injury, rest and compression are in themselves insufficient to produce a cure, continue the state of immobility in which you have placed the limb, and apply blisters, moxas, iodine, or some other stimulant to the seat of fracture.

3. If both of these modes fail in producing a deposition of callus, employ frictions.

4. If the methods mentioned fail to produce a change, or, the patient has already been suffering from his injury for eight or ten months, and there is no contraindication to it, resort to the seton.

5. If the case be one to which, from its long standing, or state of the injured parts, the seton is inapplicable, expose the fracture, and apply caustic potash to the fractured ends.

6. If all the above means have been carefully resorted to unsuccessfully, and not till then, resect the ends of the bone.

7. Never resort to amputation of the member until fair trials have been made with all these methods, and then only at the request of the sufferer, after he has found that the limb can be of no possible service to him.

In employing any of the above means, the obstacle to the occurrence of union which may exist, arising from the state of the constitution, should be carefully sought for and combated by an appropriate treatment."

Then follows a tabular statement of one hundred and fifty cases of ununited fracture, carefully compiled from American, English and French journals and surgical works, in which is recorded the age and sex of patient, bone fractured, period it had existed, operation, accidents which followed, length of time under treatment, results, and methods of cure which had previously failed. The essay closes with the following summary:

"Of the above one hundred and fifty cases of ununited fractures—

Forty-eight occurred in the femur, of which thirty-one were cured, nine without benefit, six died, and two result not stated.

Thirty-three occurred in the leg, of which thirty-two were cured, and one without benefit.

Forty-eight occurred in the humerus, of which thirty-one were cured, fourteen without benefit, and three died.

Nineteen in ihe forearm, of which seventeen were cured, one without benefit, and one died.

Two in the jaw, both of which were cured.

Of forty-six cases in which the seton* was employed, thirty-six were cured, three partial cures, five no benefit, and two died.

Of thirty-eight cases in which resection† was employed, twenty-four were cured, one partial cure, seven no benefit, and six died.

Of thirty-six cases in which pressure and rest were employed, twenty-nine were cured, one partial cure, and six no benefit.

Of eight cases in which caustic was employed, six were cured, and two derived no benefit.

Of eleven cases in which frictions were employed, all were cured.

Of eleven cases in which other methods‡ were employed, seven were cured, one received no benefit, two died, and one result not stated.

The results in the preceding table exhibit, probably, with tolerable accuracy, the success of the seton and resection, though not of the other methods of treatment, which, being milder, were in several of the cases employed before the two just named and more severe ones were resorted to. Thus it would appear as if all the cases treated by frictions had been cured, whereas, in fact, in the thirty-six cases cured by the seton, frictions had been unsuccessfully tried in eight of them; and in the twenty-four cases cured by resection, they had been equally unavailing in five of them. This will be seen by referring to the table, but could not be exhibited in the summary without complicating it more than we desired.

Of one hundred and twelve cases in which the age is noted, there were—

Between ten and twenty years of age, fourteen; between twenty

*Including the methods of Weinhold, Somme, Openheim, and Seerig.

†Including all cases in which the ends of the bones were scraped, rasped, or excised.

‡Iodine, 3, all cured; Injections, 1, cured; Erysipelas, 1, cured; Hot iron, 1, cured; Amputation, 5, 1 cured, 2 died, 1 failed, 1 not stated.

and thirty, fifty-three; between thirty and forty, twenty-one; above forty, twenty-four.

From the tables and summary, the following conclusions may be drawn:

1. That non-union after fractures is most common in the thigh and arm.

2. That the mortality after operations for its cure follows the same laws as after amputations and other great operations upon the extremities, viz.: that the danger increases with the size of the limb operated on, and the nearness of the operation to the trunk; the mortality after them beirg greater in the thigh and humerus than in the leg and forearm.

3. That the failures after operations for their relief are most frequent in the humerus.

4. That after operations for the cure of ununited fractures, failures are not more frequent in middle-aged and elderly than in younger subjects.

5. That the seton and its modifications is safer, speedier and more successful than resection or caustic.

6. That incising the soft parts previous to passing the seton augments the danger of the method, though fewer failures occur after it.

7. That the cure by seton is not more certain by allowing it to remain for a very long period, while it exposes to accidents.

8. That it is least successful on the femur and humerus."

The next essay is "*On the Treatment of Deformities following Unsuccessfully Treated Fractures.*" Under this head is discussed those irregularly united fractures, which are sometimes attended with so much shortening or deformity as to render the limb unsightly, painful, or altogether useless.

He suggests for the removal of such deformities three modes: The first, to straighten the crooked limb by means of well applied pressure; the second, to refracture the bone at the point of former injury, in order, by an after treatment, to give it a better direction; the third consists in making a section of or removing the projecting or angular portion of bone which gives rise to the deformity. Each mode is thoroughly discussed with a report of numerous cases illustrating the different plans of treatment.

The chapter on Dislocations, Fractures and Compound Fractures, in addition to the tabular statement of over two thousand fractures, and one hundred and seventy-five dislocations, gives a report in detail of quite a number of cases illustrating interesting or peculiar features of these injuries, as noted in patients received at the Pennsylvania Hospital.

In his discussions of amputations, he simply gives a tabular statement of four hundred and twenty-eight cases in which is recorded the name, age, disease or injury requiring the amputation, part amputated, immediate or otherwise, results, period of discharge or death, with the following summary of results:

"Of 428 amputations upon 424 patients, performed during the thirty years from 1830 to 1860, 321 were cured, and 103 died. Of these, 261 were primary, of which 54 died; 83 were secondary, of which 31 died; 84 were for the cure of chronic diseases, of which 18 died.

One hundred and ninety-four of the amputations were of the upper extremity, of which 21 died; 234 were of the lower extremity, of which 74 died; 46 were amputations at the joints, of which 6 died.

One hundred and eighteen of the patients operated on were under 20 years of age, of whom 108 were cured, and 10 died; 133 were between 20 and 30, of whom 101 were cured, and 32 died; 87 were between 30 and 40, of whom 60 were cured, and 27 died; 62 were between 40 and 50, of whom 40 were cured, and 22 died; 21 were upwards of 50, of whom 16 were cured, and 5 died."

The chapter on "*Statistics of the Mortality following the Ligature of the Arteries*," embraces the subclavian, iliac, carotids and femoral arteries. He gives a tabular statement of sixty-nine cases in which the subclavian artery was ligated, either within the scaleni or below the clavicle. Fifty-six were ligated for the cure of aneurism, nine in consequence of wounds or secondary hemorrhage, one for rupture of axillary artery in an attempt to reduce an old laxation, and three were done for diseases supposed to be aneurismal. Of the sixty-nine cases, thirty-six recovered, and thirty-three died.

Of the one hundred and eighteen cases of ligation of the iliac arteries included in the tabular statement, ninety-seven were done for the cure of aneurism, eighteen in consequence of wounds or secondary hemorrhage, three for the cure of varicose aneurisms; eighty-five recovered, and thirty-three died.

Of the carotid artery, he reports thirty-eight cases where this vessel was tied for the cure of aneurisms. Of this number, twenty-two recovered and sixteen died. Of thirty cases in which the artery was ligated to arrest hemorrhage from wounds, fifteen recovered, and fifteen died. In eighteen cases where the common carotid artery was tied previous to or at the time of the extirpation of tumors of the neck or face, six died, and eleven recovered; one result not known. Of six cases where the carotid was ligated for cerebral affections, all recovered from the operation, but the majority were but little, if at all, benefitted by the ligation. In forty-two cases, the carotid was ligated with the view of arresting the flow of blood to erectile tumors of diploœ, jaw, maxillary sinus and neck. Of this number twenty were cured, thirteen died, and nine recovered from the operation, though not cured by it.

Brasdor's operation was performed on fifteen cases for the cure of aneurisms of the root of the carotid artery, or arteria innominata. Of this number, four appear to have been cured, six recovered from the operation with some relief of the symptoms after it, four died and one, in which the artery was perhaps not ligated, there was no improvement.

Of the femoral artery, his tables contain two hundred and four cases where this artery was ligated, either for aneurisms or other causes. The artery was tied one hundred and eighty-three times for the cure of aneurisms, of which one hundred and forty-two were cured, and forty-six died.

Of twenty-one cases ligated for other causes, four died.

As before stated, it is a valuable contribution to practical surgery, and should have a prominent place in every medical library.

*A Treatise on Acology and Therapeutics, with some of the most Prominent Principles and Rules of Chemical and Mechanical Pharmacy.* By J. G. Westmoreland, M. D., Professor of Materia Medica and Therapeutics in Atlanta Medical College. Atlanta, Ga.: Plantation Publishing Company's Press. 1873. Pp. 391.

We are indebted to M. Lynch & Co., booksellers, Whitehall street, for a copy of a book of the above title.

Every conscientious medical man, when called upon to give a dispassionate opinion of the merits and demerits of a text-book, upon any branch of his profession, ought to enter upon the task with a proper estimate of the responsibility involved.

Text-books for the student ought to be prepared with the most conscientious care and judgment. Monographs, being addressed to those presumed to be learned in the profession, may abound in erroneous theory and even misstatements of facts. The readers for whom they are intended, are in general capable of detecting and exposing the errors they contain.

But text-books for students, being intended as foundations, upon which professional knowledge shall be based, may do incalculable harm, if the principles they inculcate are erroneous, or the facts stated in them are false *in toto*, or so stated as to obscure or too highly to color the truth.

Upon opening the volume before us, and noting its title, the author's name, and its imprint, it was with no little embarrassment that we undertook a critical examination of it, with the intention of complying with the request of the editor of the JOURNAL, to put in print our candid opinion of it. The fact that the author is a friend and associate of many years, a native of Georgia, and a resident of Atlanta, and that the book is the first, intended strictly as a text-book for medical students, that, in the knowledge of the writer, has ever been published south of Philadelphia, are considerations tending naturally to bias the mind in the direction of a favorable judgment. But justice to an experienced and successful teacher of the particular branch of medical science of which the volume is proposed as a text-book, demands a frank expression of the merits of the work before us. Apposite to a fair judgment of its merits, we note the plain, concise language in which it is written, the natural arrangement of its contents, and the complete index, whereby convenience of reference is secured.

The author seems to have directed his labors in preparing his book, to the end of furnishing a compendium of principles and facts, so arranged and classified as to furnish the student with rudimental knowledge of the Materia Medica and their therapeutic values and uses, of such a character as he can systematically add to in the study, and appropriate in the practice of the healing art, in accordance with correct principles.

With much evidence of painstaking in the effort to condense into a reasonably small space the essential principles and facts bearing upon the department of medicine, of which he proposes to furnish a text-book, the author is generally explicit, and rarely, if ever, obscure.

The novel feature of the book is the author's classification of the Materia Medica, in which it can be safely asserted, that he has better succeeded in conforming to the physiological basis than any one who has preceded him in the effort.

That the aim of the author, in his effort to classify remedies in accordance with their physiological action, may be comprehended, the following quotations are made:

"Eberle, Pereira and Wood take nominally the physiological action of remedies for the basis of their arrangement; but from their tables the learner cannot obtain a clear idea of a complete system." * * * * * * * * * *

"In the arrangement we have adopted, harmony, in many particulars, with the systems referred to, will be discovered. To these authors, particularly the great American Pharmacologist, Dr. Geo. B. Wood, the director of our studies in early life are we indebted for much that will be found in the pages of this work; not only concerning the properties of remedies and their arrangement into classes, but for views connected with their *modus operandi* and application in the treatment of the disease."

"To carry out the strictly physiological classification, new names must necessarily take the place of those, by which classes of remedies are generally known. This innovation is pardonable as the means of consistency in arrangement and great practical benefit to the student."

We leave the readers of the book, who are familiar with previously proposed classifications, to compare them with the simple and easily understood system proposed by Dr. Westmoreland. To have perfected the latter must have required much labor and thought on the part of the author. To the writer of this notice its simplicity seems to be its chief recommendation. No one but an experienced teacher, who has faithfully labored in the class-room, could ever have been induced to bestow so much thought as Prof. W. has evidently done, upon a subject usually considered dry by the majority of students.

The conscientiousness of the author is evident on every page of the book; and while it pretends only to be a *primer* to be used in the study of Acology and Therapeutics, it is a monument of educational skill, not less honorable to the author, than would be a book, abounding in details of original investigations. For he, who can arrange and present for study scientific information already diffused, in a form most easily grasped, and capable of easy application to useful purposes, is no less entitled to the thanks of mankind, than he who devoted to original investigations develops new truths.

Every student, who faithfully stores in his mind the contents of the Compendium, herein noticed, will, we opine, have no reason in future life to regret that at his initiation in medical science, he had met with this unpretending book.

The typography, paper and binding of the book we have never seen surpassed, and are highly creditable to the Plantation Publishing Company of Atlanta, from whose press and bindery it issues. The paper is of the best, few typographical errors occur, and every sentence seems to have been so carefully pondered, that it would be hypercritical to point out blemishes or defects so few and so manifestly accidental as are those we have been able to discover. S. H. STOUT, M.D.

"*Fistula, Hæmorrhoids, Painful Ulcer, Stricture, Prolapsus, and other Diseases of the Rectum, their Diagnosis and Treatment.* By WILLIAM ALLINGHAM, Fellow of the Royal College of Surgeons of England; Surgeon to St. Mark's, Hospital for Fistula, &c.; Late Surgeon to the Great Northern Hospital. Second edition, revised and enlarged. Philadelphia: LINDSAY & BLAKISTON, 1873.' From the publishers.

The above work, comprising 265 pages, comes to us from the publishing house of Lindsay & Blakiston, Philadelphia, being the second edition, and from the hands of its author so late as December, 1872.

In the notice to this second edition, he says: "The exceedingly favorable criticisms of the medical press, at home and abroad, and the rapid sale of this work, lead me to believe that in publishing it, I supplied a want felt by my professional brethren.

"I have endeavored to correct errors, and make this edition more useful, without greatly increasing it in size. The additions relate chiefly to the questions of treatment." The author has acted upon the opinion that "a big book is a big evil;" and right well has he "compressed the matter contained in this volume into the smallest compass consistent with clearness." The author writes in a plain, straightforward style, and gives the result of his extensive experience in the treatment of rectal diseases, rather than the theories that may have been promulgated by others or entertained by himself. He opens the work with an "analysis of 4,000 consecutive cases observed by himself in the out-patients' department of St. Mark's Hospital," showing the relative proportions of the several diseases to which the rectum is subject; while in the body of the work he takes them up in the order of their predominance in his analytical table, and dwells upon them in proportion to their importance, or prevalence, in their simple and complicated forms, illustrating each with well-condensed reports of cases, giving treatment and results. We say, "illustrating," there are neither wood cuts nor lithographs to illustrate the work, but the author's pen photographs are drawn so natural, that one, to follow his description, cannot fail to recognize the disease or the case he describes, while there are no unnecessary pen-strokes in the picture.

In treatment he is decidedly conservative, though when the nature of the case imperatively demands it, he strikes boldly. The spirit of the following sentiment, from the preface, pervades the body of the work.

"Though rectal operations are among the most satisfactory in the whole range of surgery, yet *I think it must be conceded that, to obviate, by judicious treatment, the necessity for an operation, is more meritorious than to perform it well when required.* (The *Italics* are our own, and we most heartily endorse the sentiment, not only in rectal, but in general surgery.)

Where the author touches upon what he conceives to be the errors of others, he deals fairly and kindly, and puts forth his own views, based upon his extended experience, in a plain, practical way, with no spirit of self-sufficiency.

We cannot venture upon any thing of a synopsis of the work, for it is a synopsis of itself, and, to be properly appreciated, must be read—*studied.* It certainly supplies a want felt by the profession; and while we would commend it to their favorable notice, we cannot refrain from expressing our regret that it contains no index to aid the busy practitioner in a ready reference to its valuable contents—a thing we ever regret, especially in works of a practical character.

In justice to the editors of this journal, we would say to the publishers, that the delay in this notice of the work was no fault of theirs, but was unintentionally caused by the reviewer. The typography and mechanical execution of the work is in the usual good style and taste of the publishers; and while we are not growing particularly old, we are happy to see that these and a few other medical publishers are beginning to realize the fact that the eyes of medical men are worth something—to ourselves, at least—and that they are sending out their publications as they have this—in plain, full-sized type.

W. A. L.

[A number of books and pamphlets have been received which will be noticed in the next number of the JOURNAL.]

